# An Excellent Selection

**Published:** 1897-08

**Authors:** 


### AN EXCELLENT SELECTION.

We are indeed very much rejoiced to announce the appointment of Prof. Leonard Pearson to the important post of Dean of the Veterinary Department of the University of Pennsylvania. We have long felt, in common with the profession, that all such places should be held by veterinarians, and while this position has been well filled in the past by Dr. John Marshall, a very earnest and true friend of the veterinary profession, a capable and efficient officer, we are still of the opinion that it has not been without its drawbacks to the institution, and that the selection of a veterinarian will add very much strength to this school in the eyes of the profession. Specially will the institution be benefited by calling to this place one so peculiarly well equipped. The happy faculties demanded for a successful man in such a place as this are more than possessed in the broadest sense by Prof. Pearson, and we look for greater strides, greater results, and greater honors for this department under his direction.

**Make this your summer outing trip, at Nashville, Sept. 7th, 8th, and 9th.**

---

Editor Tait Butler, of the *Southern Farm Gazette*, very forcibly criticises the dangerous and ignorant advice of one "Shep," who poses in the *Breeders' Gazette* as one capable of answering all questions as to treatment of diseases of sheep. The *Breeders' Gazette* is too good a paper to tolerate such exhibitions of ignorance and presumption in its columns.

---

The Journal has received many very warm and appreciative comments of Messrs. Cary, Reynolds, and Connoway bulletins, for all of which we are proud.

